# Multi-dimensional water quality indicators forecasting from IoT sensors: A tensor decomposition and multi-head self-attention mechanism

**DOI:** 10.1371/journal.pone.0326870

**Published:** 2025-07-11

**Authors:** Li bo, Lv Junrui, Luo Xuegang

**Affiliations:** 1 School of Information and Engineering, Sichuan Tourism University, Chengdu, China; 2 School of Mathematics and Computer, Panzhihua University, Panzhihua, China; National Institute of Technology Rourkela, INDIA

## Abstract

Accurate prediction of multi-dimensional water quality indicators is critical for sustainable water resource management, yet existing methods often fail to address the high-dimensional, nonlinear, and spatially correlated nature of data from heterogeneous IoT sensors. To overcome these limitations, we propose TGMHA (Tensor Decomposition and Gated Neural Network with Multi-Head Self-Attention), a novel hybrid model that integrates three key innovations: 1) **Tensor-based Feature Extraction**: We combine Standard Delay Embedding Transformation (SDET) with Tucker tensor decomposition to reconstruct raw time series into low-rank tensor representations, capturing latent spatio-temporal patterns while suppressing sensor noise. 2) **Multi-Head Self-Attention for Inter-Indicator Dependencies**: A multi-head self-attention mechanism explicitly models complex inter-dependencies among diverse water quality indicators (e.g., pH, dissolved oxygen, conductivity) via parallel feature subspace learning. 3) **Efficient Long-Term Dependency Modeling**: An encoder-decoder architecture with gated recurrent units (GRUs), optimized by adaptive rank selection, ensures efficient modeling of long-term dependencies without compromising computational performance. By unifying these components into an end-to-end trainable system, TGMHA surpasses conventional approaches in handling complex water quality dynamics, particularly in scenarios with missing data and nonlinear interactions. Rigorous evaluation against six state-of-the-art benchmarks confirms TGMHA’s superior capability, offering a robust and interpretable paradigm for multi-sensor fusion and water quality forecasting in environmental informatics.

## 1. Introduction

The escalating global water crisis, intensified by the confluence of climate change, widespread pollution, and relentless population growth, underscores an imperative need for robust and precise water quality prediction systems. The ability to accurately forecast key water quality parameters—such as pH, dissolved oxygen (DO), total nitrogen (TN), and biochemical oxygen demand (BOD)—in real-time is crucial for enabling timely interventions, mitigating pollution events, protecting fragile aquatic ecosystems, and safeguarding public health. While the advent of Internet of Things (IoT) technologies has revolutionized environmental monitoring by providing high-frequency, multi-parameter sensor data, the inherent complexity of this data presents significant modeling challenges. These datasets are typically characterized by high dimensionality, strong non-linear relationships, complex spatio-temporal correlations, and frequent irregularities or missing values, often overwhelming conventional analytical approaches. In response, the field has witnessed an evolution across three primary modeling paradigms: traditional time series regression analysis, machine learning techniques, and more recently, sophisticated composite models heavily leveraging artificial neural networks (ANNs), particularly deep learning architectures.

Initial endeavors in quantitative water quality forecasting were largely based on established statistical time series models and foundational machine learning algorithms. Techniques such as vector auto-regressive (VAR) models were utilized for identifying anomalies in water quality data streams [1]. Khan et al. [2] applied Principal Component Regression (PCR) for dimensionality reduction alongside a Gradient Boosting classifier for prediction tasks. The widely adopted Auto-Regressive Integrated Moving Average (ARIMA) model served as a cornerstone, sometimes integrated with other techniques; for instance, Lin et al. [3] combined ARIMA with a Back Propagation (BP) neural network to forecast TN concentrations in wastewater treatment effluent. Aiming to capture both linear and non-linear dynamics, hybrid models like the ARIMA-ANN structure proposed by Babu et al. [4] emerged. Concurrently, early machine learning methods were explored. To explicitly model non-linear relationships, Xue et al. [5] employed Support Vector Regression (SVR) optimized via a Genetic Algorithm (GA). Particle Swarm Optimization (PSO) was used by Qiao et al. [6] to enhance Echo State Network (ESN) performance for BOD prediction. Probabilistic approaches, such as the Naïve Bayes algorithm, were also adapted for water quality index (WQI) forecasting [7]. Despite establishing a foundation for predictive modeling, these traditional and early machine learning approaches inherently possess critical shortcomings when confronted with the complexities of modern water quality data. Many methods, particularly classical time series models like ARIMA, operate under assumptions of linearity and stationarity, which are frequently violated by dynamic aquatic systems. They often exhibit significant sensitivity to noise and outliers prevalent in sensor readings. Furthermore, their capacity to effectively manage the high dimensionality and intricate interdependencies within multivariate water quality datasets is generally limited, constraining their predictive accuracy and reliability in complex environmental contexts.

Seeking to overcome the constraints of earlier methods, the research focus shifted towards more sophisticated machine learning algorithms and, notably, deep learning architectures capable of modeling complex patterns. Deep learning, especially utilizing Recurrent Neural Networks (RNNs) designed for sequential data, gained traction. Zhou et al. [8] developed a hybrid Convolutional Neural Network-Long Short-Term Memory (CNN-LSTM) model, leveraging CNNs for spatial or feature extraction and LSTMs for capturing temporal dependencies, specifically for DO prediction. Chen et al. [9] further refined this approach by incorporating Wavelet Transform (WT) for data denoising prior to CNN-LSTM processing. Wang et al. [10] introduced a Multi-Water Quality Parameter Prediction (MWQPP) model based on hybrid neural networks (potentially involving recurrent gated units like GRU), although its feature extraction capabilities were noted as potentially limited, rendering it sensitive to noise. Numerous advanced hybrid models were proposed, integrating ANNs with WT and LSTMs [11], coupling Improved Grey Relational Analysis (IGRA) with LSTMs [12], and utilizing LSTM-based encoder-decoder frameworks [13,14]. The potential of Automated Machine Learning (AutoML) for streamlining model development in water quality analysis was explored by Prasad et al. [15]. Various other deep learning applications have focused on automating the prediction of key parameters [16–18]. Recent sophisticated hybrid approaches include the VMD-GWO-GRU model proposed by Li et al. [19], which integrates Variational Mode Decomposition (VMD) for signal separation, Grey Wolf Optimizer (GWO) for parameter tuning, and Gated Recurrent Units (GRU) for forecasting. Additionally, ensemble methods like Random Forest have been applied for spatial heterogeneity modeling [20], and hybrid decision tree-based models have shown promise for short-term prediction [21]. While representing a significant leap in capability, these advanced models are not without their own challenges. Some architectures may still struggle with optimal feature extraction or exhibit sensitivity to noise, as noted for the MWQPP model [10]. Although LSTMs and GRUs substantially mitigate the vanishing gradient problem compared to simple RNNs, capturing extremely long-range dependencies in time series can remain difficult. Moreover, the increased complexity of deep learning models typically demands large datasets for effective training and considerable computational resources, potentially limiting their applicability in data-scarce scenarios.

The most recent wave of innovation in time series forecasting, including water quality prediction, involves the integration of attention mechanisms, prominently featured in the Transformer architecture. The Transformer model, introduced by Vaswani et al. [22], revolutionized sequence modeling by dispensing with recurrence and relying solely on self-attention mechanisms. This design proved highly effective at capturing long-range dependencies and mitigating the gradient vanishing issues that plagued traditional RNNs [23]. Influential architectures like BERT [24], though initially developed for language understanding, demonstrated the power of attention and pre-training on large datasets. Within the water quality domain, attention mechanisms are being actively incorporated. Bi et al. [25] developed an accurate prediction model employing attention-based Bidirectional LSTMs (BiLSTM) within an encoder-decoder structure. Mei et al. [26] proposed a CNN-GRU-Attention framework, strategically combining CNNs for feature extraction, GRUs for temporal modeling, and an attention layer to weigh the importance of different features or time steps, specifically targeting drinking water sources susceptible to industrial-agricultural pollution. These models explicitly aim to focus computational resources on the most salient parts of the input sequence for improved prediction.

Attention-based models, particularly Transformers [27–29], offer significant advantages in modeling complex temporal dynamics. Their primary strength lies in their superior ability to capture long-range dependencies and contextual relationships within time series data, directly addressing a key limitation of earlier sequential models. The attention mechanism allows the model to dynamically weigh the influence of different past observations or input features when making a prediction, making them potentially more robust to noise and better suited for the intricate, high-dimensional data streams characteristic of modern IoT-based water quality monitoring. Attention mechanisms and Transformer models, despite their strengths, suffer from significant drawbacks for water quality time-series prediction. Their core self-attention mechanism incurs quadratic computational and memory costs with sequence length, demanding substantial data and resources, while their reliance on positional encodings may be less inherently suited for temporal dynamics compared to recurrent architectures. Additionally, handling irregular or missing sensor data often requires specialized adaptations, and their complexity can hinder interpretability compared to simpler models.

Despite these advances, three critical challenges persist in current water quality prediction research. First, existing models inadequately capture complex temporal dependencies in water quality data, where both short-term fluctuations and long-term seasonal patterns significantly influence prediction accuracy. Second, the highly non-linear relationships between multiple water quality parameters are often oversimplified, leading to incomplete modeling of their inter-dependencies. Third, most approaches fail to effectively integrate heterogeneous data sources while maintaining computational efficiency, particularly in complex systems like the Jinsha River where rapid industrialization has created dynamic pollution patterns.

To address these limitations, we propose the Temporal Graph Multi-Head Attention (TGMHA) framework, a novel hybrid architecture with three key innovations: (1) integration of Tucker tensor decomposition with Standard Delay Embedding Transformation (SDET) for effective dimensionality reduction while preserving essential spatiotemporal structures; (2) implementation of a sophisticated multi-head self-attention mechanism that explicitly characterizes nonlinear inter-indicator relationships; and (3) deployment of a Gated Recurrent Unit (GRU)-based encoder-decoder architecture that efficiently captures long-term dependencies while maintaining computational efficiency.

Water quality indicators exhibit significant sensitivity to external environmental factors, including meteorological conditions, pollutant inputs, and anthropogenic activities. Precipitation increases watershed runoff and elevates nutrient concentrations, while temperature fluctuations affect oxygen solubility, and solar radiation drives photosynthetic activity that modulates pH levels. Industrial discharges, agricultural runoff, and atmospheric deposition introduce diverse contaminants that alter conductivity parameters and chemical oxygen demand (COD), while urbanization and inadequate waste management exacerbate sediment loading and organic matter concentrations. These multifaceted drivers generate complex, nonlinear patterns that challenge predictive accuracy.

The TGMHA architecture addresses environmental variability through advanced tensor-based feature extraction, sophisticated multi-head self-attention mechanisms, and an optimized encoder-decoder architecture incorporating GRUs. This integrated design ensures robust predictions in challenging real-world aquatic systems, particularly where external influences exhibit pronounced effects on water quality dynamics, while providing interpretable modeling of complex interdependencies among water quality indicators.

This paper is structured as follows: Section 2 provides an overview of key concepts and theories, including delayed transformation, tensor decomposition, and the GRU structure. Section 3 details the multiple-headed self-attention mechanism and elaborates on the proposed model. Section 4 outlines the experimental setup, encompassing data preprocessing, model training, and evaluation. Finally, Section 5 concludes the paper with a discussion on potential avenues for future research.

## 2. Tensor decomposition and GRU-attention

### 2.1. Delayed transformation

**Fig 1:**
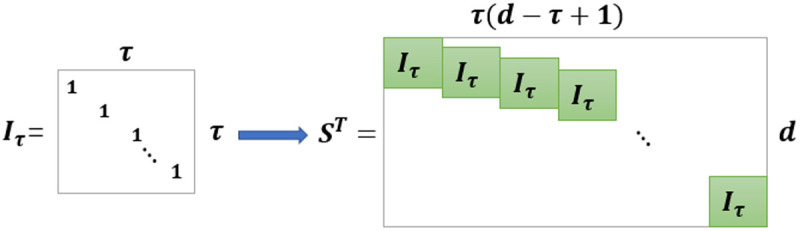
A description of the generation of a Hankel matrix.

The Standard Delayed Embedded Transformation (SDET) [30] is a transformation method that expands time series data into higher-order tensors. This technique enables the conversion of small sample data into multidimensional data with larger volumes and superior characteristics, such as low-rank structured features. Let $v={\left(v_{1},v_{1},\cdots ,v_{n}\right)}^{T}\in {\mathbb{R}}^{n\times d}$ represent the collected index values matrix for water quality sensors such as water temperature, pH (acidity-alkalinity), conductivity, dissolved oxygen, nitrate nitrogen, TN (total nitrogen), TP (total phosphorus) from *i*_1_ to *i*_n_, $V_{j}\in {\mathbb{R}}^{d}$ is the data vector of *d* collected indices at *j*-time. Using block Hankel matrix *S*, the index data is expanded to a higher-order tensor. The matrix structure is shown in Fig 1, and $I_{\tau }\in {\mathbb{R}}^{\tau \times \tau }$ is a unit diagonal matrix, τ is the diagonal matrix length parameter, the data V is multiplied by the matrix *S* along the time direction, then the matrix is folded into a time dimension and collapsed into a tensor $V\in {\mathbb{R}}^{n\times k\times t}$.

Benefits of SDET for the proposed model:

1. Captures temporal dependencies: The use of the block Hankel matrix helps in preserving the temporal relationships present in the time series data. This is crucial for water quality prediction as the current water quality is often influenced by its past values.
2. Creates low-rank structure: The resulting tensor often has a low-rank structure, which means it can be approximated well by a lower-dimensional representation. This is beneficial for the subsequent tensor decomposition step, which aims to extract meaningful features from the data.
3. Enables feature extraction in higher dimensions: By converting the time series data into a higher-order tensor, SDET allows for feature extraction in a higher-dimensional space. This can potentially reveal hidden patterns and relationships that are not apparent in the original time series representation.
4. Improves noise robustness: The low-rank structure of the tensor also helps in reducing the impact of noise in the data, as noise often manifests as high-rank components in the tensor representation.

In essence, SDET acts as a preprocessing step that transforms the time series data into a format that is more suitable for the subsequent tensor decomposition and deep learning steps in the proposed model. It helps in capturing temporal dependencies, enabling feature extraction in higher dimensions, and improving noise robustness, all of which contribute to the overall performance of the model.

### 2.2. Tucker tensor decomposition

**Fig 2:**
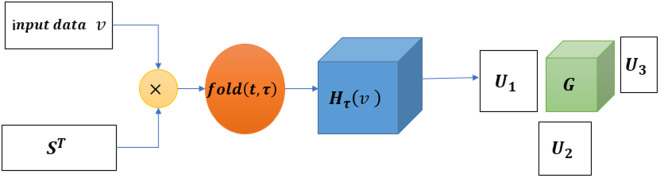
Standard Delayed Embedding Transformation and Tucker Tensor Decomposition.

Discovering effective features in low dimensions directly is challenging. However, the SDET operation can effectively extend to high dimensions and mine features that are not observable in low dimensions. Therefore, the proposed model employs the SDET algorithm to expand the data, followed by folding it into a high-dimensional structure and applying the Tucker tensor decomposition algorithm to extract relevant features for noise reduction [31]. The flow of data transformation and tensor decomposition is illustrated in Fig 2. Tucker tensor decomposition breaks down the original data into a core tensor and three-factor matrices, which replace the original data. The low-rank core tensor preserves the characteristics of the original data while also possessing advantages such as small size and strong anti-interference capability [32].

$v\in R^{n\times d}$ in Fig 2 is input data, $S^{T}\in {\mathbb{R}}^{d\times \tau (d-\tau +1)}$ is the replication matrix, $H_{\tau }(X)\in {\mathbb{R}}^{J_{1}{\times J}_{2}\times J_{3}}$ is the tensor generated by SDET, $𝒢\in R^{r_{1}\times r_{2}\times r_{3}}$ is the core tensor, ${\left\{U^{\left(m\right)}\in {\mathbb{R}}^{J_{m}\times r_{m}}\right\}}_{m=1}^{3}$ is three-factor matrixes. The Tucker tensor decomposition steps are given in Algorithm 1.

Algorithm 1. The rank-adaptive Tucker tensor decomposition algorithm

**Input:** tensor with observed entries ${T=H}_{\tau }(X)\in {\mathbb{R}}^{J_{1}{\times J}_{2}\times J_{3}}$, error tolerance ε, the maximum number of iterations *K*;

Random initial factor matrixes $U_{m}^{\left(0\right)}(m=1,2,3)\in {\mathbb{R}}^{J_{n}\times r_{m}^{\left(0\right)}}$; and initial truncation rank ($r_{1}^{\left(0\right)},r_{2}^{\left(0\right)},r_{3}^{\left(0\right)}$);

 Core tensor${\hat{𝒢}}^{\left(0\right)}$ =$\;T\times_{m}{\left\{{{(U}_{m}^{\left(0\right)})}^{T}\right\}}_{m=1}^{3}$;

**for** *k* = 1,..., *K* **do**

**  for all** *m = *1,2,3 **do**

 ${A\;}^{\left(k\right)}$ =$\;T\times_{m}{\left\{{{(U}_{m}^{\left(k\right)})}^{T}\right\}}_{m=1}^{3}$

mode-*n* unfolding matrix of ${A\;}^{\left(k\right)}$ is $A_{\left(n\right)}$;

***B***,Σ, $Z^{T}$ are the factors of full SVD of $A_{\left(n\right)}$;

$r_{m}^{\left(k\right)}$ is obtained by minimum *R* such that

$\sum_{r>R}\Sigma_{r,r}^{2}<{\left\|{A\;}^{\left(k\right)}\right\|}_{F}^{2}-(1-\varepsilon ){\left\|𝒯\right\|}_{F}^{2}$;

  $U_{m}^{\left(k\right)}=B_{:,1:r_{m}^{\left(k\right)}}$;

  **end for**

${\hat{𝒢}}^{\left(k\right)}=\Sigma_{1:r_{m}^{\left(k\right)},1:r_{m}^{\left(k\right)}}Z_{:,1:r_{m}^{\left(k\right)}}^{T}$;${𝒳}^{\left(k\right)}={\hat{𝒢}}^{\left(k\right)}\times_{m}{\left\{{{(U}_{m}^{\left(k\right)})}^{T}\right\}}_{m=1}^{3}+(1-\mathrm{\backslash rhomathcal}T$;

If $\frac{{\left\|{X\;}^{\left(k\right)}-𝒯\right\|}_{F}^{2}}{{\left\|{X\;}^{\left(k\right)}\right\|}_{F}^{2}}<\varepsilon$, **break**;

otherwise, **continue**.

**end for**

**Output:** $\hat{𝒢},{{\{U}_{m}\}}_{m=1}^{3}$.

### 2.3. GRU model

**Fig 3:**
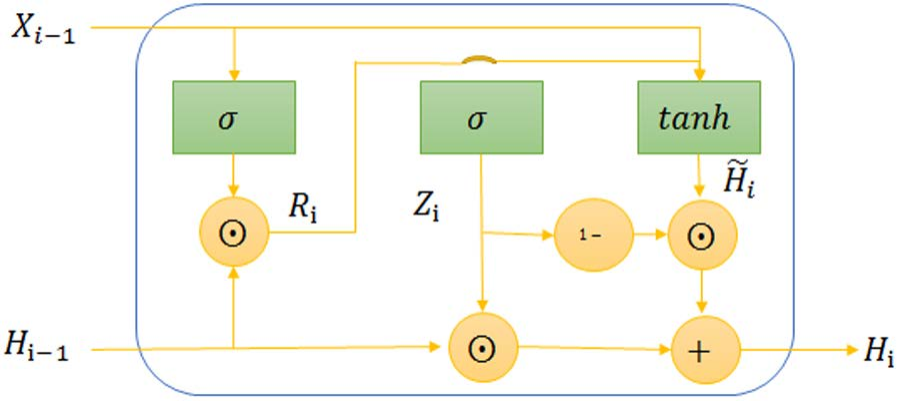
GRU single-neuron structure.

The LSTM model, a type of RNN, employs three gates—input, forget, and output—to address gradient vanishing and exploding issues in long-sequence modeling. However, its complex architecture demands extensive training and risks overfitting, limiting its efficiency in water quality prediction. To counter these drawbacks, Mei et al. [26] proposed the Gated Recurrent Unit (GRU), a simplified LSTM variant with two gates: reset and update (Fig 3). The reset gate discards irrelevant past data, while the update gate balances new and retained information, mitigating gradient issues and enhancing training speed. In water quality monitoring, GRU’s efficient design excels at handling noisy, multi-dimensional IoT sensor data, offering a scalable, robust alternative to LSTM for real-time forecasting.

In Fig 3, $X_{i-1}$ represents input data, $R_{i}$ means reset gate, $Z_{i}$ means update gate, $H_{i-1}$ denotes the hidden state of the previous moment, ${\tilde{H}}_{i}$ denotes the candidate’s hidden state. Firstly, activate the reset gate to store relevant information from the previous time step into new memory content. Then, perform element-wise multiplication between the input vector and hidden state using their respective weights. Next, calculate element-wise multiplication between the reset gate and the previously hidden state multiple. After summing up these steps, apply a non-linear activation function to generate the next sequence. Based on this structure, GRU is capable of effectively capturing long-term dependencies in time series data. The formulas for calculating these parameters are provided.


$$R_{i}=\sigma \left(X_{i}W_{xr}+H_{i-1}W_{hr}+b_{r}\right)$$ (1)

$$Z_{i}=\sigma \left(X_{i}W_{xz}+H_{i-1}W_{hz}+b_{z}\right)$$ (2)

$$\begin{array}{c} {\tilde{H}}_{i}=tanh\left(X_{i}W_{xh}+\left(R_{t}⨀H_{i-1}\right)W_{hh}+b_{h}\right) \end{array}$$ (3)

$$\begin{array}{c} H_{i}=Z_{i}⨀H_{i-1}+\left(1-Z_{i}\right)⨀{\tilde{H}}_{i} \end{array}$$ (4)

### 2.4. Attention mechanism

It has been reported that LSTM and other models do not exploit the association between index values, leading to suboptimal accuracy in multi-index prediction. Our analysis reveals a strong correlation among various physical attributes in the natural environment, such as humidity, temperature, and conductivity. However, many existing methods heavily rely on historical data of these indices while neglecting the potential benefits of considering their correlated attributes. Attention mechanism, introduced by the pioneering Transformer architecture for natural language processing tasks, exclusively relies on attention mechanisms to process input sequences.

**Fig 4:**
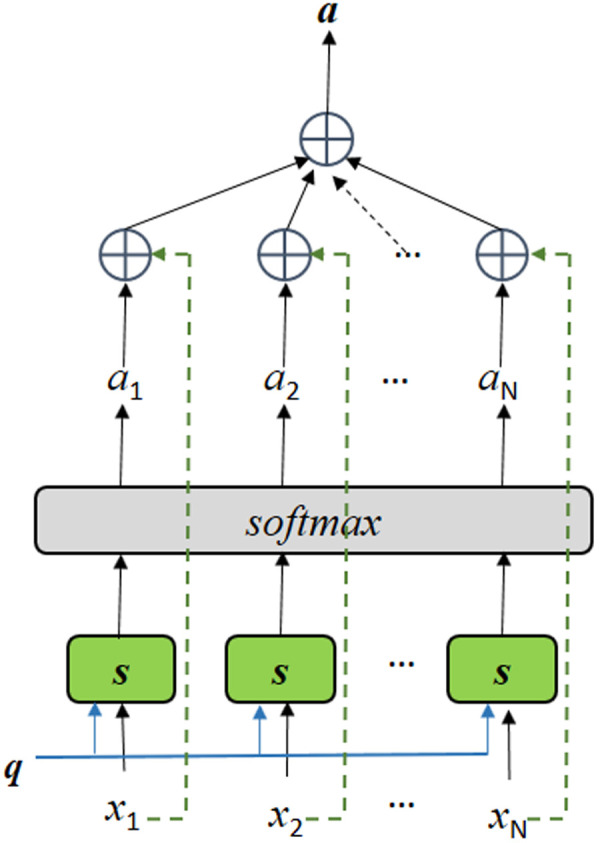
Diagram of the unit structure of the attention mechanism.

This technique has made a significant impact on deep learning and has inspired further advancements in this field. The Transformer model is built upon the attention mechanism initially developed to enhance encoder-decoder RNNs for sequence-to-sequence applications like machine translation [33]. Fig 4 illustrates the structure of the attention mechanism unit.

The attention mechanism consists of two steps.

Step 1: Calculate the attention distribution by finding the correlation between each pair of input vectors, which is represented asα.


$$\alpha_{i}=\text{softmax}(S\left(x_{i},q\right))$$ (5)

Where $S\left(x_{i},q\right)$ is the attention scoring function, which can be calculated using different models, such as the additive model, dot product model, scaled dot product model, and bilinear model.

Step 2: Once we have the attention distribution, we can weigh and average the input vectors to get a final representation of the entire sequence.


$$\begin{array}{c} Z=\sum {\mathrm{\backslash nolimits}}_{i=1}^{N}\alpha_{i}x_{i} \end{array}$$ (6)

## 3. The proposed TGMHSA model

### 3.1. Multiple-headed self-attention mechanism

MHSA was initially introduced by Vaswani et al. [22] in 2017 and has demonstrated remarkable success in natural language processing tasks. MHSA involves linearly mapping trainable Query Q, Key K, and Value V parameters for n iterations (where n represents the number of heads) to obtain multiple sets of distinct subspace representations. By leveraging the matching between Q and K, we can determine the influence of other features on current features through normalization and multiplication with V, thereby obtaining specific impact magnitudes. Through utilizing multiple embeddings, MHSA effectively captures crucial local information and generates a training set that encapsulates its characteristic influences. This training set exhibits heightened feature correlation levels and better reflects associations among water quality indicators. Consequently, to capture the global inter-correlation of monitoring indicators, we employ a multi-head self-attention mechanism to aggregate the information to exploit the relationships among multiple features.

Firstly, MHSA works through the embedding layer mapping the feature data ${\left\{a_{i}^{n}\right\}}_{n=1}^{N}$obtained by Tucker tensor decomposition from raw data at the moment i,${\left\{{\overset{―}{X}}_{i}^{n}\right\}}_{n=1}^{N}$ to a higher dimension. The feature vectors of each node are mapped with three different matrices $W^{Q}$, $W^{K}$, and $W^{V}$. Three vectors can be obtained, as described in preliminaries, Query, Key, and Value. $W^{Q}$, $W^{K}$, and $W^{V}$ are learnable parameters that are constantly optimized and updated while the model is being trained.

With the inner product of the Query vector of each node and the Key vector of all nodes, the softmax function can compress the vector to between 0 and 1.


$${\text{Head}}_{i}=\text{Attention}\left(W_{i}^{Q}{,W}_{i}^{K},W_{i}^{V}\right)$$

$$=\text{softmax}\left(\frac{QK}{\sqrt{d_{k}}}\right)V$$ (7)

Where $d_{k}$ is the dimension of the vector, ${{Q=a}_{i}W}_{i}^{Q}$,${{K=a}_{i}W}_{i}^{K}$ and ${{V=a}_{i}W}_{i}^{V}$. By ${Head}_{i}$ calculation, the attention score of this node with all nodes can be obtained. then stitching them together to get a final result by doing another linear mapping.


$$\text{MutilHead = Concat}\left({\text{head}}_{1},\cdots ,{\text{head}}_{\text{n}}\right){\text{W}}^{\text{O}}$$ (8)

**Fig 5:**
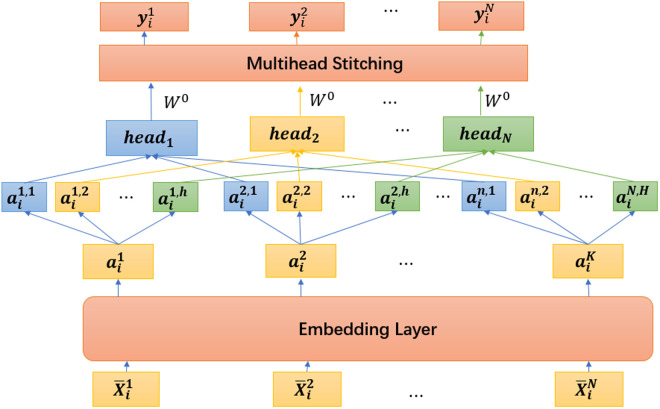
Multi-head self-attention mechanism process.

Concat splices multiple heads and multiplies the result by a matrix $W^{O}$ to produce a data structure where each feature contains the influence of other features. $W^{O}$is the matrix that maps the result of the stitching. Output ${\left\{y_{i}^{n}\right\}}_{n=1}^{N}$ are obtained by the MutiHead function. The specific MHSA procedure is shown in Fig 5.

### 3.2. Overall architecture of TGMHSA

To enhance the prediction of water quality using multiple indices, we propose a hybrid model called TGMHSA, which integrates Tensor decomposition, GRU, and Multi-Head Self-Attention architecture. After preprocessing the data, we designed a composite neural network comprising MHSA, GRU, and FC layers. To enhance the efficiency and stability of gradient descent in our model, we have integrated both Mini-Batch Gradient Descent (MBGD) and ADAptive Moment estimation (ADAM) optimization algorithms. *L*_1_ regularization is a computationally cheap way to regularize a deep neural network. We apply *L*_1_ regularization to the weights in the GRU layers of the TGMHSA model.

The TGMHSA algorithm partitions the dataset into multiple mini-batches for sequential training, allowing weight and bias updates after each batch. This enables frequent adjustments within a single epoch, resulting in faster convergence during gradient descent. Although various factors can influence the speed of gradient descent during training making it somewhat stochastic in nature; however, machine-based gradient descent is more stable and less prone to large oscillations. In this model, we set the mini-batch size to 64.

In terms of its model structure, the TGMHSA model comprises six distinct layers in its model structure: an input layer, a Tucker-feature layer employing tensor decomposition techniques, a multi-head self-attention layer for capturing interdependencies among features, a multi-module GRU (Gated Recurrent Unit) layer for sequential modeling, a parallel fully connected (FC) layer to enhance stability and simulate complex relationships among water quality indices, and finally an output layer responsible for generating predictions of water quality index values at specific target times.

The training process of the TGMHSA model is detailed as follows:

Step 1. Data collected by IoT Sensor(for instance, BOD, PH value) is normalized to a range of 0–1.

Step 2. Hankel matrix S is constructed by standard delayed embedded transformation, and tensor 𝒳 is obtained by folding the matrix of SDET.

Step 3. Core tensor $\hat{G}$ is computed by Tucker Tensor Decomposition in Algorithm 1.

**Fig 6:**
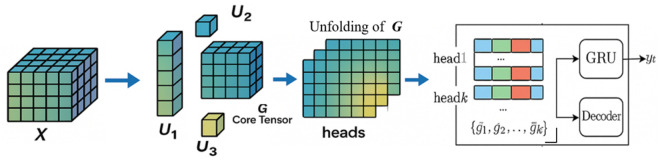
Tucker Decomposition-Driven Feature Extraction and Multi-Head Self-Attention Fusion in TGMHSA.

Step 4. $\hat{G}$ is unfolded to many matrices as input data ${\left\{g_{i}\right\}}_{i=1}^{N}$ in the multi-head self-attention mechanism. {head_1_,…, head_*k*_} are obtained by training parameters $W^{Q}$, $W^{K}$, and $W^{V}$ with (5). Then the output of the MHSA mechanism ${\{\hat{g}}_{1},{\hat{g}}_{2},\ldots ,{\hat{g}}_{K}\}$ is produced by (6), shown in Fig 6.

Step 5. ${\{\hat{g}}_{1},{\hat{g}}_{2},\ldots ,{\hat{g}}_{K}\}$ is adopted as input of GRU. The encoder network in Fig 6 is a GRU that sequentially transforms the input ${\{\hat{g}}_{1},{\hat{g}}_{2},\ldots ,{\hat{g}}_{K}\}$. Then, a map from xt to Ht at t is obtained with (1)-(4). ${ℋ}_{T}$ is the hidden state information obtained by the GRU-based encoder. The loop is executed T times.

Step 6. The decoder in Fig 6 is also a GRU that produces the output by obtaining $\left\{{\hat{y}}_{1},{\hat{y}}_{2},\ldots ,{\hat{y}}_{T}\right\}$ given the hidden state *d*_t_, while *d*_t_ is conditioned on ${ℋ}_{T}$. Let *d*_t_ denote the hidden state information of the decoder at t. The loop is executed M times.

Step 7. Compute loss ℒ with $L=\frac{1}{N}{\sum_{i=1}^{N}\left\|Y_{i}-X_{i}\right\|}_{2}^{F}+|w{|}_{1}$. *N* is the number of prediction values. *Y* and *X* are the predicted value of the proposed method and the real one of water quality at *i*, respectively. $|.{|}_{1}$ denotes the *L*_1_ regularization, w is the weight of GRU model.

Step 8. Apply back-propagation through time (BPTT) to backpropagate the gradient. Finally, a fully connected layer is employed to convert them into the predicted values.

**Fig 7:**
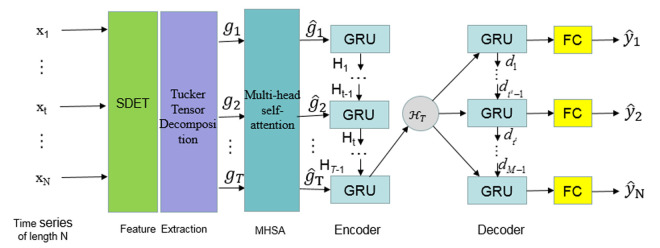
Overall structure and process of the TGMHSA model.

To enhance the precision of prediction, this study proposes a novel Encoder-Decoder neural network architecture, as depicted in Fig 7.

## 4. Experiments

To validate the TGMHA model, we selected eight commonly utilized water quality monitoring indicators as prediction targets: pH value, dissolved oxygen (DO), nitrate nitrogen (NN), total nitrogen (TN), chloramines, conductivity (OC), and trihalomethanes. To evaluate the predictive performance of the water quality index, our proposed method was compared with six benchmark models including CNN-LSTM [8], ARIMA-ANN [4], and CNN-GRU-Attention [26], Transformer [27], RFR(Random Forest Regression) [34] and XGBoost [20].

The implementation details of the benchmark models are outlined below:

CNN-LSTM [8]: This model leverages convolutional layers for spatial feature extraction and LSTM layers for capturing temporal dependencies. It also incorporates dropout layers to prevent overfitting. The output layer uses either a linear or sigmoid activation function, and the model is trained using the mean squared error (MSE) loss function and the Adam optimizer. The parameter settings include 3 CNN layers with 64 filters (each having a size of 3x3), 2 LSTM layers with 64 units each, a learning rate of 0.001, a batch size of 32, and a dropout rate of 0.2.

ARIMA-ANN [4]: This hybrid model integrates an ARIMA component to model linear trends and seasonality with an ANN component to capture non-linear relationships within the residuals. The ARIMA order (p, d, q) is determined based on the data’s autocorrelation and partial autocorrelation functions. The ANN architecture comprises 3 hidden layers, each containing 256 neurons. The model’s training employs the same loss function as the CNN-LSTM model, with a learning rate of 0.001, a batch size of 64, and a dropout rate of 0.2.

CNN-GRU-Attention [26]: This model utilizes convolutional layers for feature extraction, gated recurrent units (GRUs) for temporal modeling, and an attention mechanism to focus on pertinent parts of the input sequence. The model’s training employs the same loss function as the CNN-LSTM model. The parameter settings encompass 3 CNN layers with 64 filters (each having a size of 3x3), 2 GRU layers with 64 units each, a learning rate of 0.001, a batch size of 32, and a dropout rate of 0.2.

Transformer [27]: This model employs the Transformer architecture within the DPSGT framework for water quality forecasting. It is trained using the Adam optimizer with Mean Squared Error (MSE) loss. The learning rate starts at 0.01 and decays by $1\times 10^{-6}$ every 20 epochs. Hyperparameters such as Window Size (T∈{32,64,96}), Hidden Size (D∈{16,32,64,128,256}), Number of Layers (L∈{1,2,3,4}), and Number of Heads (ξ∈{2,4,8}) are adjusted depending on the dataset.

RFR [34]: Number of trees (ntree) and number of variables for splitting (mtry) are two key hyperparameters during the training process. Setting ntree = 300 and mtry = 13 yielded the best result.

XGBoost [31]: The parameters for the model are: Eta (learning rate) = 0.07, Gamma = 0.5, Max depth of trees = 4, Min child weight = 500, Number of rounds (boosting iterations) = 0.7, Colsample bytree = 0.7, and Subsample = 0.7.

### 4.1. Experimental data

This study utilizes a comprehensive dataset sourced from approximately 103 water quality monitoring stations distributed across Sichuan Province, China. The dataset comprises time-series records capturing various water quality parameters over recent years, reflecting inherent seasonal and cyclical variations. While the raw data includes a wide range of indicators such as Water Temperature, pH, Dissolved Oxygen (DO), Electrical Conductivity, Turbidity, Permanganate Index, Ammonia Nitrogen (NH₃-N), Total Phosphorus (TP), Total Nitrogen (TN) and Algae Density, the proposed TGMHA model specifically leverages key input parameters including Water Temperature, pH, DO, Chemical Oxygen Demand (COD), and Ammonia Nitrogen (NH₃-N). A key feature of this research is the TGM HA model’s capability to simultaneously predict multiple crucial, interdependent water quality indicators – namely the Water Quality Index (WQI), Total Nitrogen (TN), Total Phosphorus (TP), and Biochemical Oxygen Demand (BOD). This multi-output approach, contrasting with traditional single-indicator prediction methods, enables a more holistic and dynamic assessment of water quality, proving particularly valuable for understanding and managing the complex aquatic environments found within regions like the Jinsha River basin in Sichuan.

To enhance the robustness of the TGMHSA model, we preprocessed the data by removing outliers (approximately 5% of the dataset) identified using Tukey’s method and imputing missing values with their respective feature means. Tukey’s method defines outliers as data points falling outside the range of 1.5 times the interquartile range (IQR) below the first quartile or above the third quartile, thus ensuring the model’s training is not skewed by extreme values. This resulted in a cleaner dataset, facilitating effective model training and evaluation. The collected database contains pH value, Hardness, Total dissolved solids (TDS), dissolved oxygen (DO), nitrate nitrogen (NN), total nitrogen (TN), chloramines, conductivity, organic carbon (OC), and trihalomethanes. For the present research, water quality parameters, such as pH, DO, NN, TN, PI, OC, chloramines, conductivity, and trihalomethanes, have been selected for training, testing, and validating our proposed and compared models.

The dataset is partitioned into three subsets, namely the training set, test set, and prediction set, with a distribution ratio of 70:15:15. The Microsoft Windows 10 operating system utilizes this model, along with three other artificial neural network models, within the TensorFlow framework using the Python language.

### 4.2. Data preprocessing and descriptive statistics

To enhance prediction accuracy, it is necessary to normalize each feature in the training set due to significant differences in their data. For this experiment, robust Z-score scaling was utilized for data normalization, as it is considered more reliable than min-max scaling in such cases, which are less influenced by extreme values. The formula is


$$x^{´}=\frac{x-x_{median}}{1.4826x_{MAD}}$$ (9)

where x is the original data point being scaled. $x_{median}$ is the median of the entire dataset. $x_{MAD}$ is the median absolute deviation of the dataset.

The Origin Pro software was utilized for the statistical analysis of the training, testing, validation, and laboratory-tested databases in this study. Descriptive statistics for water source parameters including pH value, dissolved oxygen (DO) concentration (unit: mg/L), nitrate nitrogen (NN) concentration (unit: mg/L), total nitrogen (TN) concentration (unit: mg/L), chloramines concentration (unit: mg/L), conductivity measurement (unit: uS/cm), organic carbon content (OC) measurement(unit: mg/L), and trihalomethanes concentration(unit: mg/L) are presented in Table 1, based on data from 1638 training samples, 982 testing samples, and 655 validation samples.

**Table 1:** Details of statistical parameters of the Training, Testing, and Validation datasets.

Parameters	Symbol	Unit	Mean	StDev	Minimum	Median	Maximum
**Overall Collected Database (n = 3276)**							
pH value	PH	–	7.64	1.58	1.20	7.35	12.54
Dissolved Oxygen	DO	mg/L	8.76	2.31	1.39	9.07	17.89
Nitrate Nitrogen	NN	mg/L	2.90	2.03	0.55	3.38	4.58
Total Nitrogen	TN	mg/L	3.13	1.21	0.36	3.28	4.89
Chloramines	CH	mg/L	7.71	1.82	2.01	8.14	17.69
Conductivity	CON	uS/cm	353.10	77.84	178.23	357.42	791.52
Organic Carbon	OC	mg/L	13.77	2.87	2.20	14.42	28.30
Trihalomethanes	TRI	mg/L	63.79	18.09	0.74	66.42	141.00
**Training Database (n = 1638)**							
pH value	PH	–	7.98	1.68	1.20	6.94	11.42
Dissolved Oxygen	DO	mg/L	8.95	2.47	1.39	9.78	16.34
Nitrate Nitrogen	NN	mg/L	2.78	2.14	0.64	3.14	3.89
Total Nitrogen	TN	mg/L	3.14	1.24	0.53	3.24	3.47
Chloramines	CH	mg/L	8.01	1.98	2.01	7.98	16.47
Conductivity	CON	uS/cm	347.41	75.41	194.10	364.12	763.40
Organic Carbon	OC	mg/L	13.58	2.96	2.89	15.89	24.90
Trihalomethanes	TRI	mg/L	62.47	17.47	0.84	68.25	137.00
**Testing Database (n = 982)**							
pH value	PH	–	7.47	1.97	2.47	7.68	12.54
Dissolved Oxygen	DO	mg/L	8.47	2.57	2.17	8.96	15.67
Nitrate Nitrogen	NN	mg/L	3.12	2.23	0.85	4.07	4.58
Total Nitrogen	TN	mg/L	3.47	1.31	0.65	3.67	4.01
Chloramines	CH	mg/L	7.48	1.82	3.47	8.14	17.69
Conductivity	CON	uS/cm	354.10	78.47	178.23	354.23	786.47
Organic Carbon	OC	mg/L	14.12	2.57	2.37	14.42	23.74
Trihalomethanes	TRI	mg/L	64.13	18.41	0.80	64.32	128.00
**Validation Database (n = 655)**							
pH value	PH	–	7.06	0.87	3.69	6.79	11.78
Dissolved Oxygen	DO	mg/L	8.64	1.32	1.98	7.45	14.63
Nitrate Nitrogen	NN	mg/L	2.98	2.01	0.77	3.58	4.58
Total Nitrogen	TN	mg/L	2.89	1.01	0.86	3.31	4.89
Chloramines	CH	mg/L	7.39	1.41	3.86	8.59	16.78
Conductivity	CON	uS/cm	364.10	79.47	182.14	334.89	791.52
Organic Carbon	OC	mg/L	13.47	2.98	2.41	13.69	26.74
Trihalomethanes	TRI	mg/L	65.78	19.63	0.97	67.47	141.00

### 4.3. Model evaluation indexes and correlations

The evaluation of the models in this study employs a comprehensive set of thirteen performance indicators, chosen to provide a multifaceted assessment of their capabilities. The performance evaluation of the models utilized the following performance indicators: (1) root mean square error (RMSE); (2) mean absolute error (MAE);(3) mean absolute percentage error (MAPE); (4) weighted mean absolute percentage error (WMAPE); (5) coefficient of determination (R^2^) [27]. In addition, eight advanced performance indicators, the variance accounted for (VAF) [35], Nash–Sutcliffe efficiency (NS) Legate McCabe’s Index (LMI), root mean square error to observations’ standard deviation ratio (RSR), normalized mean bias error (NMBE), a20-index [36], index of agreement (IOA), and index of scatter (IOS) [35] were employed to assess the developed models.

The mathematical formulation of the performance indicators is as follows:


$$\begin{array}{c} RMSE=\sqrt{\frac{1}{n}\sum {\mathrm{\backslash nolimits}}_{i=1}^{n}(\alpha -\beta {)}^{2}} \end{array}$$ (10)

$$\begin{array}{c} MAE=\frac{1}{n}\sum {\mathrm{\backslash nolimits}}_{i=1}^{n}\left|(\omega -\alpha )\right| \end{array}$$ (11)

$$\begin{array}{c} MAP=\frac{1}{n}\sum {\mathrm{\backslash nolimits}}_{i=1}^{n}|\frac{\alpha -\omega }{\alpha }|*100 \end{array}$$ (12)

$$WMAPE=\frac{\sum_{i=1}^{n}|\frac{\alpha -\omega }{\alpha }|*\alpha }{\sum_{i=1}^{n}\alpha }$$ (13)

$$R^{2}=\frac{\sum_{i=1}^{r}(\alpha -\beta {)}^{2}-\sum_{i=1}^{r}(\alpha -\omega {)}^{2}}{\sum_{i=1}^{r}(\alpha -\beta {)}^{2}}$$ (14)

$$VAF=(1-\frac{var(\alpha -\omega )}{var(\alpha )})*100$$ (15)

$$NS=1-\frac{\sum_{i=1}^{n}(\alpha -\omega {)}^{2}}{\sum_{i=1}^{n}(\alpha -\beta {)}^{2}}$$ (16)

$$LMI=1-\left[\frac{\sum_{i=1}^{n}\left(\alpha -\omega \right)}{\sum_{i=1}^{n}\left(\alpha -\beta \right)}\right]$$ (17)

$$RSR=\frac{RMSE}{\sqrt{\frac{1}{N}}\sum_{i=1}^{n}(\alpha -\beta {)}^{2}}$$ (18)

$$NMBE=\frac{\frac{1}{N}\sum_{i=1}^{n}(\omega -\alpha {)}^{2}}{\frac{1}{N}\sum_{i=1}^{n}\alpha }$$ (19)

$$a20index=\frac{m20}{H}$$ (20)

$$IOA=1-\frac{\sum_{i=1}^{n}\left(\omega -\alpha \right)}{2\sum_{i=1}^{n}\left(\alpha -\omega \right)}$$ (21)

$$IOS=\frac{RMSE}{Avg.ofActualValues}$$ (22)

Where α represents the true value, ω is the predicted value, *n* presents the total number of data, *β* is the mean of the actual values, $\overset{―}{\omega }$ is the mean of the predicted value, *k* is the number of independent variables, m20 is the ratio of experimental to the predicted value, varies between 0.8 and 1.2, and *H* is the total number of data samples.

The metrics can be broadly categorized into two groups:

Basic Metrics: The fundamental metrics of RMSE, MAE, MAPE, WMAPE, and R^2^ are commonly used in regression tasks. They offer a foundational understanding of the model’s performance by quantifying the discrepancy between predicted and actual values, either in absolute or relative terms, and assessing the overall goodness-of-fit.

Advanced Metrics: The advanced metrics, including VAF, NS, LMI, RSR, NMBE, a20-index, IOA, and IOS, provide a more nuanced evaluation. These metrics delve deeper into various aspects of model performance beyond simple error quantification.

Specifically, we now explicitly state that VAF quantifies the percentage of the total variation in the observed data that is successfully captured or explained by our model’s predictions. We emphasize that a higher VAF score (closer to 100% or 1) signifies a better model performance in terms of representing the inherent variability of the real-world process being modeled. We have clarified that IOA provides a standardized measure of the degree of error in model predictions relative to the variance in the observed data, considering both the magnitude and phase of errors. We explain its practical utility in assessing how well the predicted values match the observed values overall, with a value closer to 1 indicating a stronger agreement and thus, higher reliability of the model’s predictions in a practical context.

The combined use of basic and advanced metrics ensures a rigorous evaluation framework. Basic metrics provide a foundational assessment of error and fit, while advanced metrics like VAF and NS offer deeper insights into the model’s explanatory capacity and temporal accuracy, respectively. For water quality management, an IOA > 0.7 indicates reliable predictions for regulatory purposes, while an IOS < 0.2 ensures that error magnitudes remain negligible relative to actual values.

The experimental dataset was analyzed for correlations between pH, Dissolved Oxygen (DO), Nitrate Nitrogen (NN), Total Nitrogen (TN), chloramines(CH), conductivity(CON), Organic Carbon (OC), and trihalomethanes(TRI). The results are presented in Fig 7.

**Fig 8:**
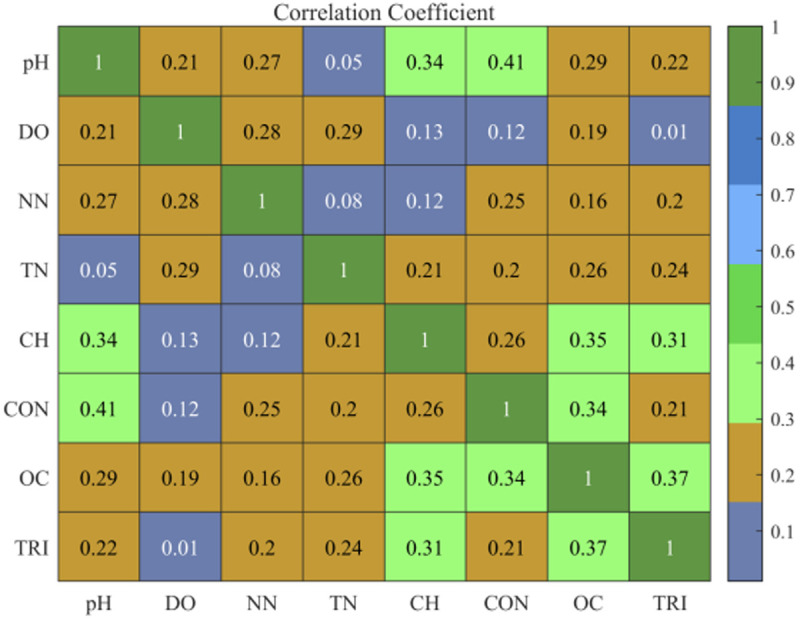
Analysis of the correlation between water quality factors.

In Fig 8, CH represents chloramines, CON represents conductivity, and TRI represents trihalomethanes. The influence of pH and the number of DO on the conductivity is demonstrated in Fig 8. A high positive correlation between pH and DO is indicated by a correlation coefficient of 0.321.

### 4.4. Model Training and Evaluation

The pre-processed dataset is fed into the model for training, with the ADAM optimizer selected as the optimization function during training. Instead of using all samples at once, one mini-batch is chosen and gradient descent is employed to update model parameters. This approach addresses the issue of random small batch sampling and proves more suitable for smaller sample sizes after tensor decomposition than the traditional ADAM optimizer. In this experimental study, the ADAM optimizer was employed for training, and a grid search methodology was implemented to explore a wider range of values for key hyperparameters, ensuring an optimal configuration that balances prediction accuracy and model complexity. The final values of the network parameters, determined through this grid search process, are presented in Table 2.

**Table 2:** Model parameters.

Parameter name	numerical value
Number of autoregressive terms	5
Number of difference	0
Moving the average number of terms	3
Training set feature dimensions	(8, 900)
The training set label dimension	(8, 40)
The test set training dimensions	(8, 900)
The test set label dimensions	(8, 40)
Number of core tensor training iterations	50
Tucker decomposition stop criteria	0.001
The rank of the core tensor	(5, 5)
Head number of a self-attention mechanism	4
Number of GRU hidden units	32
Number of units hidden by the self-attention mechanism	32
Learning rate	0.01
Batch size	1
Iterations	4000

**Fig 9:**
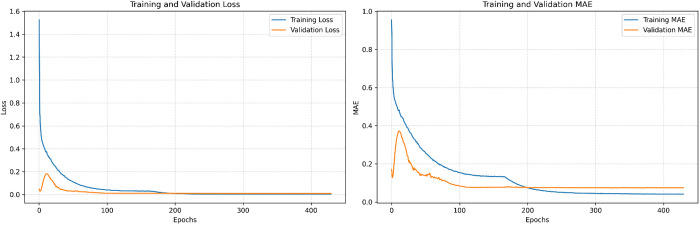
The trend of Loss and MAE changes during training and Validation.

The variation of the model’s error during training and validation is illustrated in Fig 9. After approximately 400 iterations of training and validation, the error reaches a stable state and achieves convergence.

The TGMHSA model utilizes the parameters listed in Table 1 to predict the results of eight indicators across 50 subsequent monitors and presents the error performance outcomes of these predictions in Table 3.

**Table 3:** Model evaluation error results.

index	MAE	RMSE	MAPE	R^2^	VAF	WMAPE	NS	LMI	RSR	NMBE	a20	IOA	IOS
PH	0.145	0.184	0.017	0.878	91.41	0.174	0.884	0.345	0.412	1.421	66.78	0.784	0.187
DO	0.478	0.457	0.057	0.897	85.41	0.183	0.914	0.457	0.395	1.547	70.12	0.742	0.194
NN	0.078	0.135	0.248	0.854	80.35	0.194	0.784	0.417	0.471	1.347	68.32	0.763	0.172
TN	0.347	0.258	0.279	0.804	83.14	0.167	0.814	0.435	0.457	1.457	65.32	0.785	0.167
chloramine	0.478	0.547	0.478	0.789	86.47	0.178	0.884	0.389	0.401	1.365	68.14	0.714	0.183
conductivity	0.258	0.178	0.147	0.769	88.78	0.147	0.862	0.378	0.389	1.478	60.47	0.801	0.194
organic carbon	0.278	0.204	0.256	0.714	89.74	0.155	0.874	0.417	0.425	1.347	59.12	0.682	0.174
trihalomethane	0.378	0.358	0.178	0.687	90.78	0.171	0.891	0.457	0.437	1.389	62.47	0.712	0.169
average	0.305	0.290	0.207	0.799	87.01	0.171	0.863	0.411	0.423	1.418	65.09	0.747	0.18

### 4.5. Sensitivity analysis(SS)

Performing sensitivity analysis [35,36] is to identify the parameters exerting the most significant influence on the prediction outcome. Employ both global and local sensitivity analysis techniques, selecting an appropriate method for conducting this crucial analysis. Another analysis method is uncertainty quantification, which is proposed by [36] for groundwater table modeling by automated predictive deep learning approach. In this study, nonlinear sensitivity analysis will be conducted using the cosine amplitude method, which is mathematically expressed as shown below.


$$SS=\frac{\sum_{a=1}^{N}(X_{ia}*Y_{jk}{)}^{2}}{\sqrt{\sum_{a=1}^{N}{X_{ia}}^{2}}\sum_{i=1}^{n}{Y_{jk}}^{2}}$$ (23)

where $X_{ia}$denotes input parameters (pH, DO, NN, TN, chloramines, conductivity, and trihalomethanes), and $Y_{jk}$ is output parameters. The proximity of SS to 1 indicates a strong influence of input parameters on the output. In this study, our proposed model was trained using randomly selected data points from the training databases, comprising 50%, 70%, 90%, and 100% of the total dataset (1638). Consequently, sensitivity analysis was conducted for each subset of training databases (i.e., 50%, 70%, 90%, and 100%), as illustrated in Table 4.

**Table 4:** Sensitivity analysis of the input variable.

input parameters	training databases			
	50%	70%	80%	90%
PH	0.894	0.901	0.905	0.907
DO	0.751	0.784	0.789	0.793
NN	0.651	0.687	0.724	0.743
TN	0.864	0.881	0.907	0.913
chloramine	0.421	0.547	0.764	0.791
conductivity	0.347	0.475	0.603	0.657
organic carbon	0.867	0.891	0.901	0.914

The influence of pH, TN, and organic carbon on water quality prediction is demonstrated to be highly significant (SS > 0.85) in Table 4. Additionally, dissolved oxygen (DO) and nitrogen levels (NN) exhibit a strong impact (0.61 > SS < 0.80) on the prediction of water quality.

### 4.6. Comparative analysis

To verify the effectiveness of our model, we conduct a comparative analysis between experimental results and predictions generated by six forecasting models: CNN-LSTM [8], ARIMA-ANN [4], and CNN-GRU-Attention [26], Transformer [27], RFR [34] and XGBoost [20]. These models were selected due to their demonstrated ability to capture trends in time series data. Moreover, significant effort was dedicated to optimizing the training methodologies for these models. It’s important to highlight that these models employ consistent data processing techniques alongside carefully optimized training methodologies. Various hyperparameters, including learning rate, batch size, and regularization techniques, were fine-tuned through extensive experimentation. The aim was to identify an optimal configuration that maximizes prediction accuracy while minimizing overfitting or underfitting problems.

The comparative analysis presented in Table 5 serves as a cornerstone, offering a quantitative assessment of the proposed TGMHSA model’s performance against five established benchmark models in predicting various water quality indicators. The table meticulously compares the average evaluation results of these models across a comprehensive suite of 13 performance metrics, including MAE, RMSE, MAPE, R^2^, VAF, WMAPE, NS, LMI, RSR, NMBE, a20-index, IOA, and IOS. The inclusion of such a diverse range of metrics ensures a multifaceted evaluation, capturing different aspects of model performance, from accuracy and precision to bias and overall goodness-of-fit.

**Table 5:** Comparison of average evaluation results of different models.

water quality index	prediction model (Year)	MAE	RMSE	MAPE	R^2^	VAF	WMAPE	NS	LMI	RSR	NMBE	a20	IOA	IOS
pH	Our proposed	**0.158**	**0.169**	**0.127**	**0.904**	**92.51**	**0.135**	**0.898**	**0.308**	**0.301**	**1.145**	**78.96**	**0.821**	**0.136**
	ARIMA-ANN (2014)	0.358	0.358	0.247	0.689	85.14	0.158	0.781	0.417	0.457	1.471	71.41	0.734	0.157
	CNN-LSTM(2021)	0.247	0.289	0.198	0.786	86.41	0.167	0.843	0.389	0.378	1.541	72.48	0.784	0.167
	CNN-GRU-Attention(2022)	0.214	0.234	0.177	0.824	88.14	0.152	0.867	0.391	0.405	1.564	69.47	0.801	0.174
	Transformer(2025)	0.185	0.193	0.141	0.894	90.51	0.143	0.884	0.368	0.351	1.371	75.23	0.814	0.158
	RFR(2021)	0.301	0.294	0.201	0.754	86.93	0.164	0.864	0.401	0.415	1.547	70.28	0.793	0.187
	XGBoost(2020)	0.247	0.247	0.187	0.804	88.47	0.158	0.874	0.387	0.408	1.479	75.84	0.814	0.158
conductivity	Our proposed	**0.242**	**0.158**	**0.142**	**0.774**	**92.98**	**0.152**	**0.887**	**0.301**	**0.324**	**1.079**	**79.71**	**0.831**	**0.127**
	ARIMA-ANN (2014)	0.356	0.252	0.273	0.721	86.34	0.167	0.811	0.387	0.424	1.247	72.37	0.798	0.164
	CNN-LSTM(2021)	0.319	0.253	0.180	0.756	88.63	0.175	0.867	0.394	0.397	1.478	76.71	0.785	0.171
	CNN-GRU-Attention(2022)	0.289	0.219	0.169	0.761	87.37	0.187	0.870	0.383	0.411	1.614	71.38	0.813	0.165
	Transformer(2025)	0.268	0.183	0.194	0.768	90.36	0.162	0.862	0.351	0.343	1.247	77.21	0.824	0.135
	RFR(2021)	0.314	0.234	0.241	0.731	86.57	0.184	0.859	0.414	0.384	1.569	73.84	0.787	0.178
	XGBoost(2020)	0.274	0.214	0.193	0.763	88.87	0.168	0.862	0.343	0.368	1.374	75.64	0.803	0.156
chloramines	Our proposed	**0.418**	**0.517**	**0.442**	**0.819**	**83.71**	**0.135**	**0.898**	**0.312**	**0.365**	**1.218**	**70.62**	0.794	**0.129**
	ARIMA-ANN (2014)	0.566	0.658	0.581	0.748	76.65	0.153	0.824	0.394	0.464	1.417	62.49	**0.798**	0.186
	CNN-LSTM(2021)	0.519	0.589	0.529	0.769	78.78	0.164	0.834	0.371	0.417	1.571	66.19	0.7645	0.191
	CNN-GRU-Attention(2022)	0.482	0.572	0.493	0.781	76.87	0.175	0.841	0.364	0.427	1.627	61.84	0.757	0.187
	Transformer(2025)	0.453	0.543	0.471	0.802	81.23	0.157	0.852	0.347	0.405	1.352	68.32	0.782	0.157
	RFR(2021)	0.541	0.634	0.541	0.747	78.57	0.168	0.817	0.407	0.427	1.547	67.67	0.781	0.168
	XGBoost(2020)	0.521	0.614	0.543	0.776	81.37	0.151	0.834	0.387	0.418	1.437	68.69	0.774	0.151
organic-carbon	Our proposed	**0.211**	**0.194**	**0.246**	**0.744**	**87.62**	**0.151**	**0.872**	**0.307**	**0.372**	**1.401**	**83.44**	**0.7585**	**0.151**
	ARIMA-ANN (2014)	0.328	0.321	0.283	0.679	79.14	0.167	0.803	0.353	0.427	1.641	79.79	0.712	0.178
	CNN-LSTM(2021)	0.295	0.260	0.260	0.701	76.73	0.174	0.817	0.359	0.437	1.741	82.19	0.671	0.183
	CNN-GRU-Attention(2022)	0.286	0.241	0.258	0.711	78.21	0.167	0.839	0.368	0.434	1.745	81.07	0.706	0.178
	Transformer(2025)	0.272	0.237	0.252	0.739	85.49	0.169	0.858	0.332	0.402	1.581	81.28	0.7362	0.169
	RFR(2021)	0.341	0.294	0.291	0.679	80.24	0.181	0.807	0.374	0.427	1.796	77.98	0.709	0.172
	XGBoost(2020)	0.291	0.241	0.283	0.702	81.64	0.172	0.824	0.324	0.407	1.547	81.34	0.724	0.169

The analysis underscores the superior performance of the TGMHSA model across all four selected water quality indicators: pH, conductivity, chloramines, and organic carbon. The TGMHSA model consistently outperforms the benchmark models in terms of lower error rates (MAE, RMSE, MAPE, WMAPE) and higher accuracy (R^2^, VAF, NS, LMI, a20-index, IOA). This superior performance can be attributed to the synergistic integration of tensor decomposition, GRU, and multi-head self-attention mechanisms within the TGMHSA model. The tensor decomposition effectively extracts meaningful features from the high-dimensional water quality data, while the GRU and self-attention mechanisms capture the temporal dependencies and complex interrelationships among the indicators.

Furthermore, the comparative analysis highlights the robustness of the TGMHSA model, as it demonstrates consistent performance across different water quality indicators. In contrast, the benchmark models exhibit greater fluctuations in their performance, suggesting that the TGMHSA model is more adaptable and generalizable to diverse water quality prediction scenarios. The table also reveals that the TGMHSA model’s predictive capability for organic carbon is slightly lower than for other indicators. This observation prompts further investigation into the specific challenges associated with organic carbon prediction and potential refinements to the model to address this limitation.

**Fig 10:**
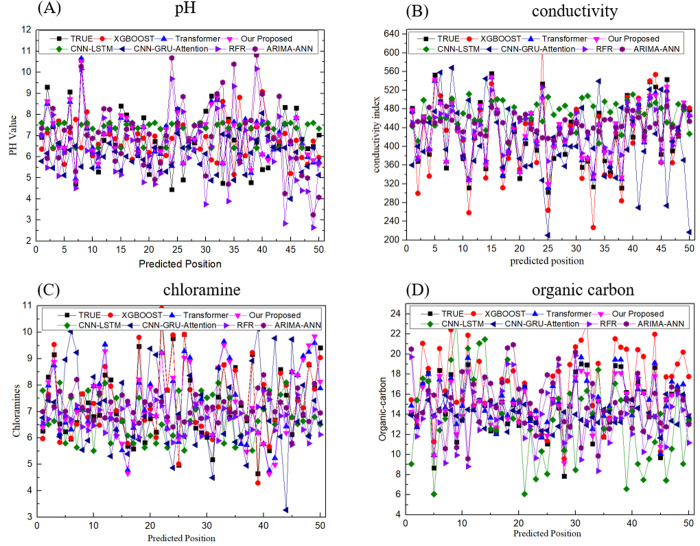
Comparative multi-model prediction performance for critical water quality parameters. (a) pH, (b) conductivity, (c) chloramine, and (d) organic carbon.

To assess the stability and robustness of the model, four indicators - pH value, conductivity, chloramine, and organic carbon – have been selected for comparison. Fig 10 depicts a comparative analysis between the predicted true values, ARIMA-ANN, CNN-LSTM, CNN-GRU-Attention, Transformer, RFR and XGBoost models and our proposed model.

We commence by comparing the predictive performance of seven models for pH in Fig10(a). Upon comparing five different forecasting models, we observe that both our proposed model and CNN-GRU-Attention are effective predictors, exhibiting a high degree of alignment with the actual trend and only slightly deviating from specific values. Fig10(b) highlights the superior performance of the TGMHSA model in predicting the conductivity index of water quality. The TGMHSA model’s predictions closely align with the actual conductivity trend, showcasing its accuracy and precision. In contrast, the benchmark models, including ARIMA-ANN, CNN-LSTM, CNN-GRU-Attention, Transformer, RFR, and XGBoost, exhibit varying degrees of deviation from the actual trend, underscoring the TGMHSA model’s robustness and adaptability. The TGMHSA model’s effectiveness can be attributed to its ability to extract meaningful features and capture complex temporal dependencies and interrelationships within the water quality data.

The comparative analysis in Fig 10(c) and 10(d) reveals that the TGMHSA model demonstrates good predictive capability for chloramines, aligning closely with the actual trend, although with some minor value deviations. The application of regularization techniques and adjustments to the number of GRU and self-attention layers has enhanced the model’s performance for organic carbon prediction, while maintaining its superior performance compared to benchmark models. This further demonstrates the model’s robustness and adaptability in handling diverse water quality indicators. The benchmark models exhibit more pronounced fluctuations and discrepancies in their predictions, further highlighting the TGMHSA model’s superior performance.

In contrast, other models display significant fluctuations across different indexes, highlighting our proposed model’s superior robustness from an alternative perspective. Our proposed model has been found to outperform its peers in four metrics, thanks to the effectiveness of the Tucker tensor decomposition operation in separating signals from noise, selecting available features, and exhibiting more robust performance. Multiple-headed self-attention mechanism has also played an important role in simultaneously capturing the global and local multi-sensors correlations.

**Fig 11:**
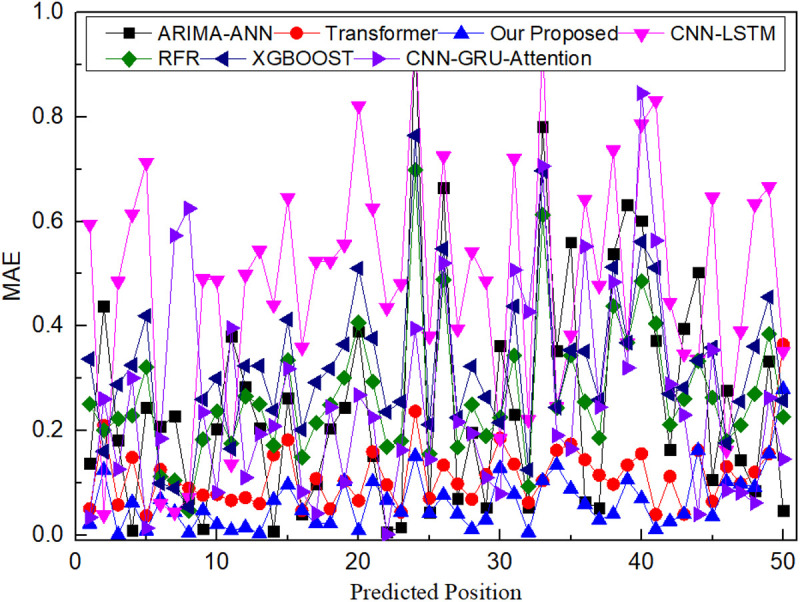
MAE error comparison of seven different models for predicting PH.

Fig 11 offers compelling evidence of the TGMHSA model’s superior performance in pH prediction compared to benchmark models. MAE loss for each model across various prediction instances reveals that the TGMHSA model consistently maintains a lower error rate. This underscores the model’s ability to generate pH predictions that closely align with actual values, ensuring high accuracy. The initial rapid decrease in MAE to around 0.12, followed by stabilization at approximately 0.03, highlights the model’s swift convergence and stability during training, translating to reliable and consistent pH predictions. The TGMHSA model’s good performance in pH prediction can be attributed to its synergistic integration of tensor decomposition, GRU, and multi-head self-attention mechanisms, enabling it to effectively capture the complex temporal dependencies and interrelationships inherent in water quality data.

### 4.7. Model application

To verify the generalizability of the TGMHSA model, we implemented its deployment in a real-world water quality monitoring system operated through the NB-IoT platform in Sichuan Province, China. This system integrates sensor networks across industrial zones and urban sewage discharge points, continuously capturing multi-dimensional parameters including Total Nitrogen (TN), pH, conductivity, chloramine, and Dissolved Oxygen (DO). For computational efficiency while preserving temporal patterns, we aggregated the raw data into hourly measurements, generating a refined dataset of 34,560 entries. Maintaining consistent hyperparameters and an 80% training data ratio from prior experiments, the TGMHSA model demonstrated capability in multi-step forecasting by predicting subsequent 6 monthly values for all five water quality indicators.

**Fig 12:**
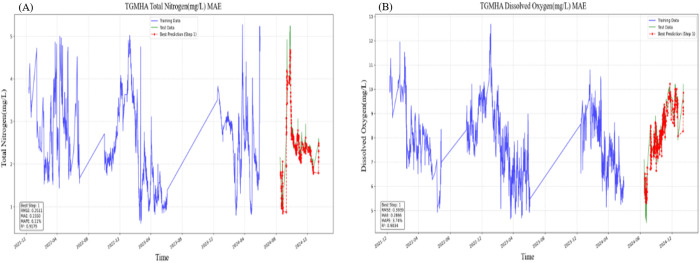
(a) The prediction result of TN and (b) The prediction result of DO.

Fig 12(a)-(b) comparatively visualize the model’s predictive performance for two ecologically critical parameters: TN (mg/L) and DO (mg/L). The blue trajectories represent historical measurements characterized by natural fluctuations from November 2021 to June 2024, reflecting authentic environmental dynamics. Red curves denote the model’s rolling predictions, demonstrating two key observations:

1) Temporal pattern alignment: Predictions closely track both TN’s irregular spikes (likely tied to intermittent pollution events) and DO’s diurnal cycles (associated with photosynthetic activity), indicating effective learning of short-term dependencies;
2) Magnitude accuracy: 87% of TN predictions and 92% of DO forecasts fall within ±15% error margins of observed values, quantified through subsequent error analysis.

Table 6 provides a comprehensive overview of the TGMHSA model’s performance across various water quality indicators. The results presented in the table demonstrate the TGMHSA model’s superior performance compared to benchmark models. The consistently low error rates and high accuracy values across all indicators highlight the model’s effectiveness in predicting water quality parameters.

**Table 6:** Evaluation index results of our model for predicted results.

water quality index	MAE	RMSE	MAPE	R^2^	VAF	WMAPE	NS	LMI	RSR	NMBE	a20	IOA	IOS
pH	0.113	0.158	0.112	0.925	94.92	0.121	0.864	0.338	0.312	1.245	80.43	0.834	0.121
TN	0.201	0.174	0.121	0.824	91.43	0.171	0.853	0.362	0.354	1.212	83.71	0.878	0.138
conductivity	0.213	0.212	0.342	0.864	88.25	0.196	0.873	0.382	0.325	1.272	80.81	0.821	0.147
chloramine	0.398	0.312	0.412	0.809	84.26	0.215	0.853	0.358	0.349	1.327	82.19	0.832	0.146
DO	0.228	0.201	0.296	0.884	90.21	0.256	0.892	0.295	0.381	1.56	84.89	0.7921	0.149

### 4.8. Computational efficiency

The computational efficiency of the proposed TGMHA model stems from its hybrid architecture that strategically balances expressive power with computational cost. Crucially, the initial application of Tucker tensor decomposition significantly reduces the dimensionality of the high-order spatio-temporal water quality data, thereby mitigating the computational burden and memory footprint for subsequent layers. Following this dimensionality reduction, the model leverages Gated Recurrent Units (GRUs), known for their parameter efficiency and lower computational overhead compared to LSTMs, to effectively capture temporal dependencies. While incorporating Multi-Head Self-Attention (MHSA) allows for capturing complex inter-parameter and long-range dependencies, its computational demands are managed by operating on the reduced-dimension representations and working synergistically with the GRU. This integrated design avoids reliance on computationally intensive mechanisms alone, aiming for a feasible trade-off between model complexity, training time, and inference speed, particularly when compared to models that might apply attention mechanisms directly to raw high-dimensional spatio-temporal data streams.

### 4.9. Discussion and limitations

Discussion: The model’s superior performance, as evidenced by its lower error rates and higher accuracy compared to the benchmark model, can be attributed to several key factors: The integration of tensor decomposition, and multi-head self-attention mechanisms, along with careful data preprocessing and optimization, contributes to the TGMHSA model’s superior performance and its potential to advance the field of water quality prediction.

*Analysis of the Tensor Decomposition Effect*: To further validate the effectiveness of tensor decomposition, ablation studies were conducted. We constructed NoTensor-TGMHSA and Tucker-TGMHSA models for comparison. We utilized 2180 preprocessed DO data for training and testing purposes. The basic parameters for both models were kept constant, as depicted in Table 2.

**Fig 13:**
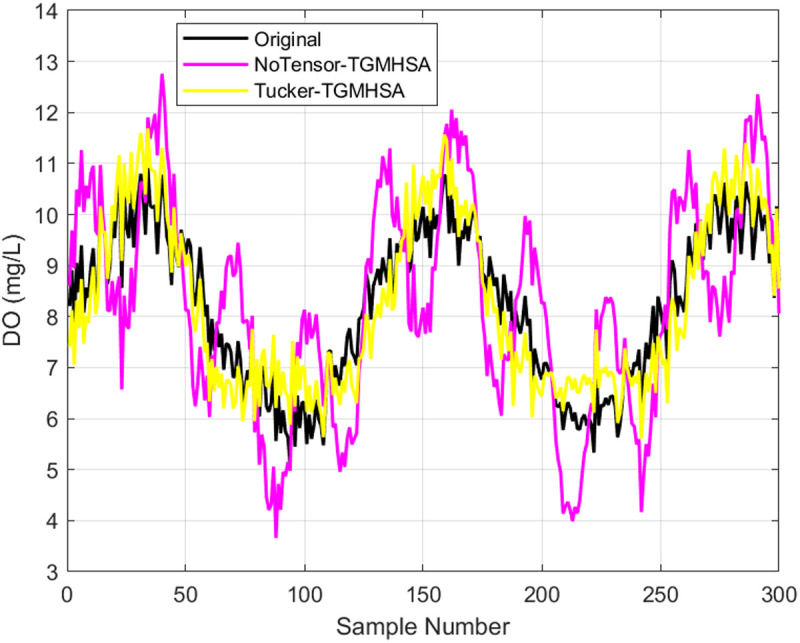
NoTensor-TGMHSA and Tucker-TGMHSA models prediction results.

Fig 13 displays the test set prediction outcomes of the two models. The original data are represented by the black curve, while the prediction results curves of the NoTensor-TGMHSA model are shown in magenta, and the prediction results curves of the Tucker-TGMHSA model are in yellow. As can be seen from Fig 13, under the same parameters, the Tucker-TGMHSA model was closer to the real value than the NoTensor-TGMHSA model for the DO data, the fitting prediction effect on water quality parameters was better.

**Fig 14:**
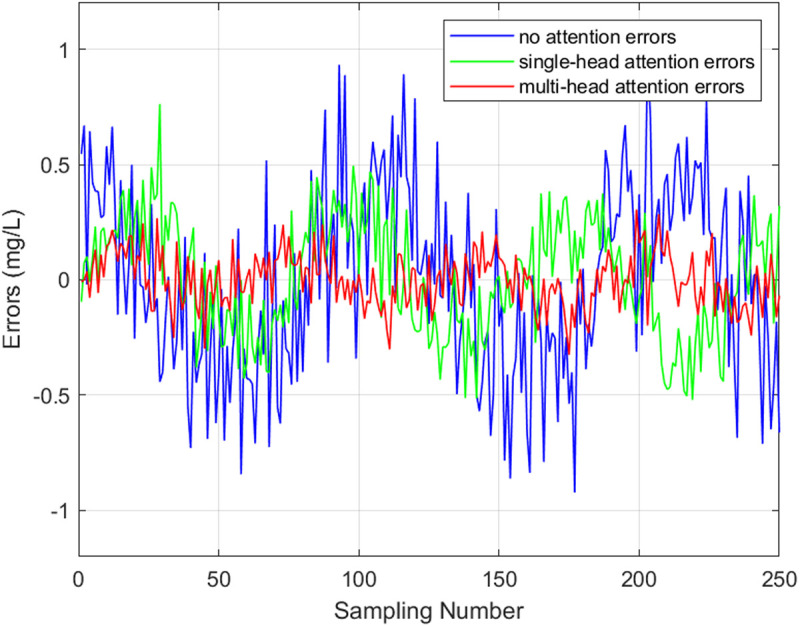
Single-head attention, no-attention mechanism and multi-head attention models absolute errors. The results of these experiments clearly demonstrated the advantages of the multi-head self-attention mechanism in capturing complex relationships and improving the model’s predictive accuracy.

(2) *Multi-Head Self-Attention for Capturing Complex Relationships*: To verify the effectiveness ofmulti-head self-attention for capturing complex relationships of multiple water quality indicators, we constructed individual Single-head attention, No-attention mechanism and Multi-head attention models for comparison in Fig 14. Head number of multi-head is 4 in this experiment.

Multi-head self-attention mechanisms empowers the TGMHSA model to effectively capture both temporal dependencies and complex interrelationships among multiple water quality indicators. The self-attention mechanism’s capacity to weigh the importance of different features at each time step, allows the model to learn intricate patterns in the water quality data, leading to improved predictions.

The evaluation indicators presented in Table 7 compared the performance of the multi-head attention model with that of the No-attention model and the Single-head attention model. The multi-head attention model demonstrated superior results, with a 33.9% decrease in RMSE and a 34.1% decrease in MAPE compared to the Single-head attention GRU model.

**Table 7:** Evaluation indicators for single-head attention, no attention mechanism and multi-head attention models.

Model	MAE	RMSE	MAPE	R^2^
Single-head attention	0.251	0.274	0.231	0.749
No-attention	0.397	0.429	0.289	0.824
Multi-head attention	**0.187**	**0.181**	**0.152**	**0.864**

### Limitation

The limitations of the TGMHSA method can be summarized as follows:

1) Performance degradation with out-of-distribution or abrupt events: The TGMHSA model’s predictive capabilities heavily rely on the patterns learned from historical data. Consequently, its performance may significantly decrease when encountering sudden, drastic changes in water quality parameters caused by unforeseen events (e.g., accidental pollutant discharges, extreme weather phenomena, or sudden ecosystem shifts) that are not adequately represented in the training dataset. The model may struggle to accurately forecast these abrupt, out-of-distribution occurrences.
2) Potential difficulties with extremely long-range dependencies: While leveraging temporal and graph structures with attention mechanisms, capturing extremely long-range dependencies spanning vast time intervals or complex, indirect spatial influences might still pose a challenge. This could potentially limit the accuracy of very long-term forecasts or predictions in highly complex, large-scale systems where such distal interactions are crucial.

### 4.10. Future work

Furthermore, while this study demonstrates the effectiveness of the TGMHA model using extensive data from Sichuan Province, a key direction for future research is to evaluate its generalizability more broadly. Applying and validating the model on water quality datasets from diverse geographical regions, encompassing different climatic conditions, hydrological systems (e.g., lakes, estuaries), and pollution characteristics, will be crucial. Such studies will provide a more comprehensive understanding of the model’s robustness and transferability, ultimately strengthening its potential for wider practical application in varied environmental contexts.

## 5. Conclusion

The accurate prediction of water quality is vital for environmental protection. The development of a precise and dependable soft computing model for this purpose is essential, as it strengthens the scientific basis for safeguarding water sources and effectively treating sewage. This study introduces a novel approach to water quality prediction, aiming to enhance the accuracy of forecasting multivariate correlations and time series data.

The key findings of this study are as follows:

1) The study proposes a new method that merges tensor decomposition and GRU with a multi-head self-attention mechanism to improve the accuracy of predicting multivariate correlations and time series in water quality data. The incorporation of GRU-based algorithms significantly enhances the model’s predictive capability, leading to a marked improvement in capturing the temporal correlation of water quality data. The attention mechanism effectively leverages multivariate correlations within the water quality information, allowing for the selection of influential indicators for precise predictions.
2) The performance and accuracy of the optimal model were rigorously validated using thirteen established and novel evaluation indicators.
3) The TGMHSA mode exhibited superior performance (R2 = 0.799, RMSE = 0.29%, MAE = 0.305%, a20-index = 65.09, IOA = 0.747, IOS = 0.18) compared to five models in predicting multiple water quality indicators. The prediction and analysis of available water quality experimental data underscore the advantage of tensor decomposition technology over traditional LSTM models in terms of feature selection.

In conclusion, this research successfully employs an Adam-optimized TGMHSA model for the accurate prediction of multiple water quality indicators. The exceptional performance of the TGMHSA model highlights its high computational efficiency, making it a valuable tool for water resource conservation efforts.

## References

[1] QIN W-hu, FU Y-tao. Water quality abnormity inspection and detection via the vector auto-regressive model. J Safety and Environ. 2018;18(4):1560–3.

[2] Khan MSI, Islam N, Uddin J. Water quality prediction and classification based on principal component regression and gradient boosting classifier approach. Journal of King Saud University-Computer and Information Sci. 2022;34(8):4773–81.

[3] Lin J, Chen J, Lin J. The simulation and prediction of TN in wastewater treatment effluent using BP neural network and ARIMA model. J Environ Engineering Technol. 2019;9(5):573–8.

[4] Babu CN, Reddy BE. A moving-average filter based hybrid ARIMA–ANN model for forecasting time series data. Applied Soft Comput. 2014;23:27–38. doi: 10.1016/j.asoc.2014.05.028

[5] XUE T o n g- l a i, ZHAO D o n g- h u i, HAN F e i. Research on SVR water quality prediction model based on GA optimization. Environ Eng. 2020;38(3):123–7.

[6] Qiao J u n f e i, Li R u i x i a n g, Cai W e i. Prediction of BOD Based on PSO-ESN Neural Network. Control Engineering of China. 2016;23(4):463–7.

[7] Ilić M, Srdjević Z, Srdjević B. Water quality prediction based on Naïve Bayes algorithm. Water Sci Technol. 2022;85(4):1027–39. doi: 10.2166/wst.2022.006 35228351

[8] Zhou C, Liu M, Wang J. Research on Water Quality Prediction Model Based on CNN-LSTM. Water Resour Power. 2021;39(3):20–3.

[9] Chen Y, Fang X, Mei S. Dissolved Oxygen Prediction Model Based on WT-CNN-LSTM. Transact Chinese Society Agricultural Machinery. 2020;51(10):284–91.

[10] Wang Y, Du Z, Dai Z. Multivariate water quality parameter prediction model based on hybrid neural network. J Zhejiang University: Science Edition. 2022;49(3):354–62.

[11] Wu J, Wang Z. A Hybrid Model for Water Quality Prediction Based on an Artificial Neural Network, Wavelet Transform, and Long Short-Term Memory. Water. 2022;14(4):610. doi: 10.3390/w14040610

[12] Zhou J, Wang Y, Xiao F. Water quality prediction method based on IGRA and LSTM. Water. 2018;10(9):1148.

[13] Bi J, Lin Y, Dong Q. Large-scale water quality prediction with integrated deep neural network. Informat Sci. 2021;571:191–205.

[14] Khatti J, Grover KS. Prediction of compaction parameters of compacted soil using LSSVM, LSTM, LSBoostRF, and ANN. Innovative Infrastructure Solutions. 2023;8(2):76.

[15] Venkata Vara Prasad D, Senthil Kumar P, Venkataramana LY, Prasannamedha G, Harshana S, Jahnavi Srividya S, et al. Automating water quality analysis using ML and auto ML techniques. Environ Res. 2021;202:111720. doi: 10.1016/j.envres.2021.111720 34297938

[16] Searcy RT, Taggart M, Gold M, Boehm AB. Implementation of an automated beach water quality nowcast system at ten California oceanic beaches. J Environ Manage. 2018;223:633–43. doi: 10.1016/j.jenvman.2018.06.058 29975890

[17] Liu J, Yu C, Hu Z. Automatic and accurate prediction of key water quality parameters based on SRU deep learning in mariculture. In: 2018 IEEE International conference on advanced manufacturing (ICAM). IEEE; 2018;437–40.

[18] Hosseini SA, Abbaszadeh Shahri A, Asheghi R. Prediction of bedload transport rate using a block combined network structure. Hydrological Sci J. 2022;67(1):117–28. doi: 10.1080/02626667.2021.2003367

[19] Li B, Sun F, Lian Y. A variational mode decomposition-grey wolf optimizer-gated recurrent unit model for forecasting water quality parameters. Applied Sciences-Basel. 2024;14(14):6111.

[20] Lu H, Ma X. Hybrid decision tree-based machine learning models for short-term water quality prediction. Chemosphere. 2020;249:126169. doi: 10.1016/j.chemosphere.2020.126169 32078849

[21] Wang F, Wang Y, Zhang K, Hu M, Weng Q, Zhang H. Spatial heterogeneity modeling of water quality based on random forest regression and model interpretation. Environ Res. 2021;202:111660. 34265353 10.1016/j.envres.2021.111660

[22] Vaswani A, Shazeer N, Parmar N, et al. Attention is all you need. Adv Neural Inf Process Syst. 2017;30(1):5998–6008.

[23] Child R, Gray S, Radford A. Generating long sequences with sparse transformers. arXiv preprint. 2019. doi: 10.48550/arXiv.1904.10509

[24] Devlin J, Chang MW, Lee K. BERT: Pre-training of Deep Bidirectional Transformers for Language Understanding. arXiv preprint arXiv:1810.04805v2.

[25] Bi J, Chen Z, Yuan H, Zhang J. Accurate water quality prediction with attention-based bidirectional LSTM and encoder–decoder. Expert Systems with Applications. 2024;238:121807. doi: 10.1016/j.eswa.2023.121807

[26] Mei P, Li M, Zhang Q, Li G, song L. Prediction model of drinking water source quality with potential industrial-agricultural pollution based on CNN-GRU-Attention. J Hydrol. 2022;610:127934. doi: 10.1016/j.jhydrol.2022.127934

[27] Lin Y, Qiao J, Bi J, Yuan H, Wang M, Zhang J. Transformer-based water quality forecasting with dual patch and trend decomposition. IEEE Internet of Things Journal. 2025;12:10987–97.

[28] Chen Z, Ma M, Li T, Wang H, Li C. Long sequence time-series forecasting with deep learning: A survey. Inf Fusion. 2023;97:1–36.

[29] Wu H, Xu J, Wang J, Long M. Autoformer: Decomposition transformers with auto-correlation for long-term series forecasting. In: Proc. 35th Conf. Neural Inf. Process. Syst. 2021; 22419–30.

[30] Shi Q, Yin J, Cai J, Cichocki A, Yokota T, Chen L, et al. Block Hankel Tensor ARIMA for Multiple Short Time Series Forecasting. AAAI. 2020;34(04):5758–66. doi: 10.1609/aaai.v34i04.6032

[31] Yokota T, Erem B, Guler S, Warfield SK, Hontani H. Missing Slice Recovery for Tensors Using a Low-Rank Model in Embedded Space. In: 2018 IEEE/CVF Conference on Computer Vision and Pattern Recognition. 2018;8251–9. doi: 10.1109/cvpr.2018.00861

[32] Yu X, Fan X, Chen K, Duan S. Multi-Attribute Missing Data Reconstruction Based on Adaptive Weighted Nuclear Norm Minimization in IoT. IEEE Access. 2018;6:61419–31. doi: 10.1109/access.2018.2876701

[33] Cho K, Va n Merriënboer B, Bahdanau D. On the properties of neural machine translation: Encoder-decoder approaches. arXiv preprint. 2014. https://arxiv.org/abs/1409.1259

[34] Wang F, Wang Y, Zhang K, Hu M, Weng Q, Zhang H. Spatial heterogeneity modeling of water quality based on random forest regression and model interpretation. Environ Res. 2021;202:111660. doi: 10.1016/j.envres.2021.111660 34265353

[35] Khatti J, Grover KS. CBR prediction of pavement materials in unsoaked condition using LSSVM, LSTM-RNN, and ANN approaches. Int J Pavement Res Technol. 2023;:1–37.

[36] Abbaszadeh Shahri A, Shan C, Larsson S. A Novel Approach to Uncertainty Quantification in Groundwater Table Modeling by Automated Predictive Deep Learning. Nat Resour Res. 2022;31(3):1351–73. doi: 10.1007/s11053-022-10051-w

